# Maternal Plasma and Amniotic Fluid Sphingolipids Profiling in Fetal Down Syndrome

**DOI:** 10.1371/journal.pone.0127732

**Published:** 2015-05-22

**Authors:** Karol Charkiewicz, Agnieszka Blachnio-Zabielska, Monika Zbucka-Kretowska, Slawomir Wolczynski, Piotr Laudanski

**Affiliations:** 1 Department of Perinatology and Obstetrics, Medical University of Bialystok, Marii Sklodowskiej-Curie 24a, Bialystok, 15–276, Poland; 2 Department of Physiology, Medical University of Bialystok, Mickiewicza 2C, Bialystok, 15–222, Poland; 3 Department of Reproduction and Gynecological Endocrinology, Medical University of Bialystok, Marii Sklodowskiej Curie 24a, Bialystok, 15–273, Poland; University Hospital Basel, SWITZERLAND

## Abstract

**Introduction:**

Sphingolipids can be potentially involved in the formation of the central and peripheral nervous systems, which are particularly connected with the pathogenesis of Down syndrome. The aim of the study was to determine the concentration of selected sphingolipids in the plasma and amniotic fluid of pregnant patients with fetal Down syndrome.

**Material and Methods:**

Out of 190 amniocentesis we had 10 patients with confirmed Down syndrome. For the purpose of our control we chose 14 women without confirmed chromosomal aberration. To assess the concentration of 11 sphingolipids in the blood plasma and amniotic fluid we used an ultra-high performance liquid chromatography coupled with triple quadrupole mass spectrometry (UHPLC/MS/MS).

**Results:**

We showed a significant increase in the concentration of 2 ceramides, C22-Cer and C24:1-Cer, in the plasma of women with fetal Down syndrome. Furthermore we showed a decrease in the concentration of 7 ceramides—C16-Cer, C18-Cer, C18:1-Cer, C20-Cer, C22-Cer, C24:1-Cer, and C24-Cer—in the amniotic fluid of women with fetal Down syndrome. We created ROC curves for all significant sphingolipids in maternal plasma, which set the threshold values and allowed for predicting the likelihood of Down syndrome in the fetus with specific sensitivity and specificity. We demonstrated a significantly higher risk of Down syndrome when the plasma concentration of C22-Cer > 12.66 ng/100ul (sens. 0.9, sp. 0.79, *P value* = 0.0007) and C24:1-Cer > 33,19 ng/100ul (sens. 0.6, sp. 0.86, *P value* = 0.0194).

**Conclusion:**

On the basis of our findings, it seems that the sphingolipids may play a role in the pathogenesis of Down syndrome. Defining their potential as biochemical markers of Down syndrome requires further investigation on a larger group of patients.

## Introduction

The incidence of Down syndrome in the United States is estimated to be 1/732 live births [1]. This syndrome is a result of a chromosomal aberration characterized by extra chromosome 21 or a fragment thereof. In people with this aneuploidy, there is a high risk of congenital heart defects, gastroesophageal reflux syndrome, sleep apnoea, thyroid disease and many other diseases [2].

Currently, the diagnosis of Down syndrome is based on non-invasive (biochemical, genetic, and ultrasound) and invasive (amniocentesis and chorionic villous sampling) prenatal test. Diagnostic efficacy of invasive method in combination with genetic diagnostics is 99.8% and they rarely give false positive results. However, these methods carry a 1% risk of miscarriage or fetal damage. In contrast, non-invasive tests themselves are connected with 5–10% false positives, and thus all positive results should be confirmed by the invasive methods [3]. A few years ago, scientists have created a non-invasive prenatal tests based on free fetal DNA present in maternal blood. These tests have a low rate of false positives, which is only 0.5%, but they are very expensive ($ 795 - $ 2762) and more available in the United States than in Europe [4–7]. Therefore, there is a need for new potential biomarkers of Down syndrome which will provide enough data for a small percentage of false positive results that will not have to be confirmed by any invasive method.

Emerging evidence suggests that chromosomal fetal aneuploidies as well as different reproductive events are under the regulatory control of different biologically active molecules, such as cytokines, growth factors and lipids, but their role in human normal and abnormal pregnancies is still largely undefined [8–11].

The rationale behind sphingolipids determination in pregnant patients with fetal Down syndrome comes from the very recent metabolomics study, which showed that specifically 3 metabolites (3-hydroxybutyrate, 3-hydroxyisovalerate, and 2-hydroxybutyrate) are significantly increased in serum of studied women. It is of interest that especially 3-hydroxybutyrate is one of the sources for the synthesis of phospholipids and sphingolipids. Both groups of compounds are essential for the process of myelination of neurons, which is considerably delayed in the brain of fetuses with Down syndrome. The scientists explain that decrease in synthesis of sphingolipids in fetal brain can result in increased level of unused 3-hydroxybutyrate in maternal blood [10, 12, 13].

Therefore, measurement of the sphingolipids in pregnancies with fetal chromosomal abnormalities could lead to better understanding of the influence of Down syndrome on such pregnancy and possibly provide new biomarker(s) for non-invasive genetic testing.

## Material and Methods

The study and control groups consisted of women who underwent routine amniocentesis between 15^th^ – 18^th^ weeks of gestation at the Department of Reproduction and Gynecological Endocrinology of the Medical University of Bialystok, Poland, (recruitment between 09.2012 and 12.2013). We performed 190 amniocentesis throughout the recruitment period.

We recruited only non febrile women without any chronic or acute disease and also excluded those taking any type of hormonal or anti-inflammatory treatment.

The study protocol was approved by the Local Ethical Committee of Medical University of Bialystok, Poland, and an informed consent was obtained from each patient (No. ethics committee approval: R-I-002/10/2014). Signed informed consent from all participants involved in the study was obtained.

We obtained 15 ml of amniotic fluid during routine amniocentesis. 10 mL of peripheral blood was collected for EDTA probes after amniocentesis from each patient. The blood was centrifuged in 15 minutes, plasma subsequently separated and frozen at −80°C temperature. After analysis of the caryotyping results, for the purpose of this study, we chose 10 women with trisomy 21 fetuses and for the control group we selected 14 healthy patients with uncomplicated pregnancies, who delivered healthy newborns at term.

The procedures are performed early in the morning and all of the patients who undergo this procedure are advised to be fasting.

The content of sphingolipids was measured using a UPLC/MS/MS in multiple reaction monitoring (MRM) mode according to Blachnio-Zabielska et al. [14]. The method uses internal standard approach with individual concentration curves prepared with the use of commercially available sphingolipid standards (Avanti Polar Lipids). Briefly, to each plasma sample (100 μl) were added 50 μl of the internal standard solution (17C-sphingosine and 17C-S1P, and C17-Cer Avanti polar lipids) as well as 1.5 ml of an extraction mixture (isopropanol:water:ethyl acetate, 35:5:60; v:v:v). The following sphingolipids were quantified: Sph (sphingosine), S1P (sphingosine-1-phosphate), SPA (sphinganine), ceramide C14:0-Cer (ceramides containing myristic acid), C16:0-Cer (ceramides containing palmitic acid), C18:1-Cer (ceramides containing oleic acid), C18:0-Cer (ceramides containing stearic acid), C20:0-Cer (ceramides containing arachidic acid), C22:0-Cer (ceramide containing behenic acid), C24:1-Cer (ceramides containing nervonic acid) and C24:0-Cer (ceramides containing lignoceric acid). Sphingolipids were analyzed by means of an Agilent 6460 triple quadrupole mass spectrometer using positive ion electrospray ionization (ESI) source with multiple reaction monitoring (MRM). Chromatographic separation was performed using an Agilent 1290 Infinity Ultra Performance Liquid Chromatography (UPLC). The analytical column was a reverse-phase Zorbax SB-C8 column 2.1 × 150 mm, 1.8 μm. Chromatographic separation was conducted in binary gradient using 2 mM ammonium formate, 0.15% formic acid in methanol as Solvent A and 1.5 mM ammonium formate, 0.1% formic acid in water as Solvent B at the flow rate of 0.4 ml/min. HPLC grade methanol, water, formic acid, ammonium formate and ethanol were purchased from Sigma-Aldrich (St. Louis, MO).

We also performed CRP (C reactive protein) determination. CRP in plasma was measured using immunoturbidimetric method with the Multigent CRP Vario assay (detectable range was 0.2–480 mg/L) detected on the ARCHITECT ci4100.

The method was validated for the use of sphingolipid estimation in biological fluids and was used for sphingolipids measurement in human plasma [15, 16]. Inter and intra assay reproducibility of the method is around 1% for most of the sphingolipid species. The list of quantified sphingolipids with the limit of detection is provided in Table 1.

**Table 1:** The list of quantified sphingolipids with the limit of detection.

Compound	LOD (fmol) on-column
d17:1 Sph	5.5
Sph	5.2
dhSph (SPA)	5.2
d17:1 S1P	4.3
S1P	4.1
C14:0-Cer	3.1
C16:0-Cer	2.9
C17:0-Cer	2.8
C18:1-Cer	2.8
C18:0-Cer	2.8
C20:0-Cer	2.6
C24:1-Cer	2.4
C24:0-Cer	2.4

Descriptive statistics including mean concentration and standard error of the mean concentration were calculated for selected sphingolipids, henceforth called features. In order to detect statistically significant differences between considered groups (Down syndrome group versus control group), either fitting an analysis of variance model [17] was conducted or non-parametric method (Wilcoxon rank-sum test [18]) was applied. The choice of an appropriate method was made upon fulfilling the normality and the homogeneity of variances assumptions and in case of violation of at least one condition non-parametric approach was employed.

The normality of features distribution was checked with the Shapiro-Wilk test [19] and the homogeneity of variances with the Levene’s test [20]. Features that have been found significant, i.e. their distribution was statistically significantly different among experimental groups, were taken under further investigation to discover their prediction capability. To accomplish that, for each significant feature: ROC curves (receiver operating characteristic curve) were constructed and optimal threshold values were determined with the Youden method [21]. Confidence intervals for sensitivity and specificity corresponding to a particular threshold were calculated with the use of the Wilson method [16] and a test verifying the area under curve (AUC), that was significantly greater than 0.5 (random classification) was performed with the DeLong method [22]—p-values and one-sided confidence intervals for AUC are reported. Calculations concerning ROC curves and corresponding tests were conducted with the functions provided by the pROC R package [23]. Confidence intervals for sensitivity and specificity were constructed with the use of the binom.confint function, part of the binom R package. All calculations were carried out in R software environment [24]. Significance level alpha equal to 0.05 was applied for all statistical tests.

## Results

Clinical characteristics of the patients are presented in Table 2. Patients from both groups were matched for maternal age, number of pregnancies, gestational age at collecting and body mass in order to make sure that two groups comparable and there are no statistically significant differences between them.

**Table 2:** Clinical characteristic of the patients.

	Group I—Down Syndrome Pregnancies (n = 10)	Group II—Pregnancies without Down Syndrome (n = 14)
Maternal age (median ± SD)	39.5 ± 8.193	37 ± 8.192
Number of pregnancies (median ± SD)	1.5 ± 0.9189	1.214 ± 1.051
Gestational age at collecting of samples in weeks (median ± SD)	15.5 ± 0.7633	16.64 ± 0.99
Present body mass in kg (median ± SD)	70.5 ± 11.04	65 ± 8.647

SD—standard deviation

The values of mean concentration and standard error of maternal plasma and amniotic fluid sphingolipids in each study group are presented respectively in Table 3 and Table 4.

**Table 3:** Concentrations of sphingolipids in maternal plasma.

	Group I—Down Syndrome Pregnancies (n = 10)	Group II—Pregnancies without Down Syndrome (n = 14)	P-value
	Sphingolipids concentration (ng/100μl) Mean ± SEM		Group I- Group II
Sph	0.5109 ± 0.1418	0.3516 ± 0.0611	0.7961
SPA	0.1882 ± 0.0505	0.2254 ± 0.0566	0.7088
S1P	31.659 ± 2.1946	28.151 ± 1.6871	0.2125
C14-Cer	0.2839 ± 0.0403	0.3024 ± 0.0254	0.6870
C16-Cer	23.622 ± 2.2615	23.437 ± 1.5547	0.9448
C18:1-Cer	0.1006 ± 0.0134	0.0947 ± 0.0059	0.6665
C18-Cer	1.4210 ± 0.1857	1.3843 ± 0.1088	0.9314
C20-Cer	8.6636 ± 1.1503	8.1356 ± 0.9683	0.7286
C22-Cer	17.759 ± 2.3913	11.477 ± 1.3829	0.0241*
C24:1-Cer	43.011 ± 7.0827	26.312 ± 3.1583	0.0265*
C24-Cer	48.697 ± 5.3932	43.627 ± 4.2294	0.4615

* statistically significant value of less than 0.05 for Student’s T-test

**Table 4:** Concentrations of sphingolipids in amniotic fluid.

	Group I—Down Syndrome Pregnancies (n = 10)	Group II—Pregnancies without Down Syndrome (n = 14)	P-value
	Sphingolipids concentration(ng/100μl) Mean ± SEM		Group I- Group II
Sph	0.1687 ± 0.0415	0.1926 ± 0.0263	0.5995
SPA	0.2609 ± 0.1535	0.0836 ± 0.011	0.5571
S1P	0.1246 ± 0.044	0.1776 ± 0.0782	0.7897
C14-Cer	0.3041 ± 0.0358	0.4096 ± 0.0384	0.0734
C16-Cer	7.9872 ± 1.1811	15.381 ± 1.8053	0.0067*
C18:1-Cer	0.0106 ± 0.0012	0.0199 ± 0.0017	0.0006*
C18-Cer	0.3282 ± 0.0385	0.7078 ± 0.0564	0.0001*
C20-Cer	2.7242 ± 0.3654	6.5533 ± 0.6422	0.0002*
C22-Cer	7.0884 ± 1.0827	14.287 ± 2.49	0.0020**
C24:1-Cer	13.204 ± 1.8057	28.280 ± 3.9798	0.0006**
C24-Cer	15.850 ± 5.7077	25.697 ± 6.7947	0.0158**

* statistically significant value of less than 0.05 for Student’s T-test

** statistically significant value of less than 0.05 for Mann Whitney Wilcoxon’s test

We showed significant increase in concentration of 2 ceramides: C22-Cer and C24:1-Cer (*P value =* 0.024 and 0.026, respectively) in plasma of women with fetal Down syndrome (Table 3). Furthermore we showed decrease in concentration of 7 ceramides: C16-Cer, C18-Cer, C18:1-Cer, C20-Cer, C22-Cer, C24:1-Cer, C24-Cer (*P value =* 0.0067, 0.0006, 0.0001, 0.0002, 0.002, 0.0006,0.0158, respectively) in amniotic fluid of women with fetal Down syndrome (Table 4).

We included all statistically significant sphingolipids in later ROC analyses but we created ROC curves only for sphingolipids significant in plasma (which has potential for noninvasive diagnosis), which set the threshold values and allowed predicting the likelihood of Down syndrome with specific sensitivity and specificity.

**Fig 1:**
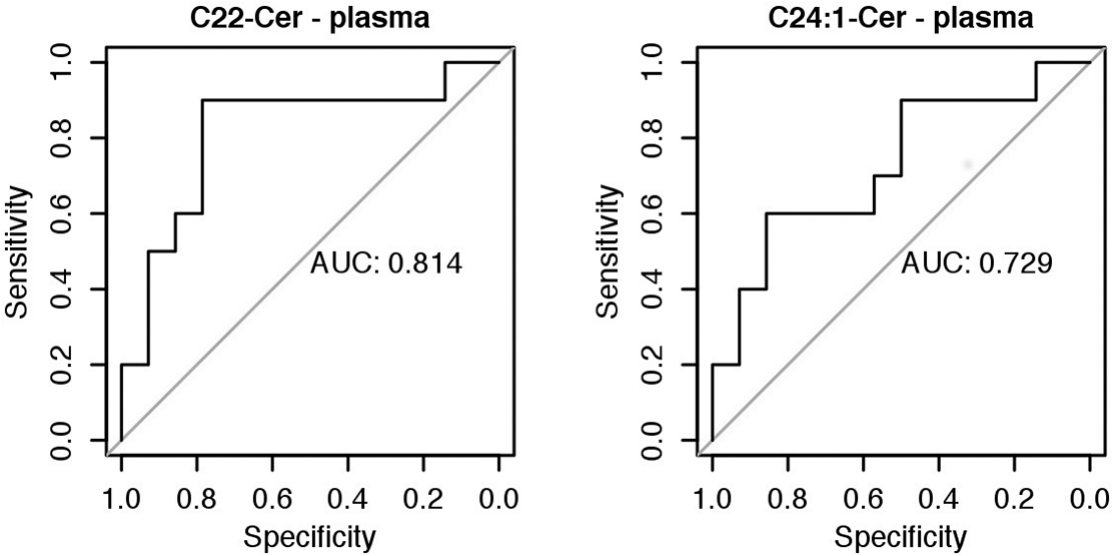
The ROC curves for concentration of sphingolipids in plasma: C22-Cer and C24:1-Cer. The area under the ROC curve for C22-Cer was 0.814 and for C24:1-Cer it was 0.729. AUC- Area under curve; ROC- Receiver operating characteristic.

The area under the ROC curve for C22-Cer was 0.814 and for C24:1-Cer it was 0.729 (Fig 1). All field values are satisfactory and indicate the usefulness of these biochemical markers as tools to predict the risk of Down syndrome.

**Fig 2:**
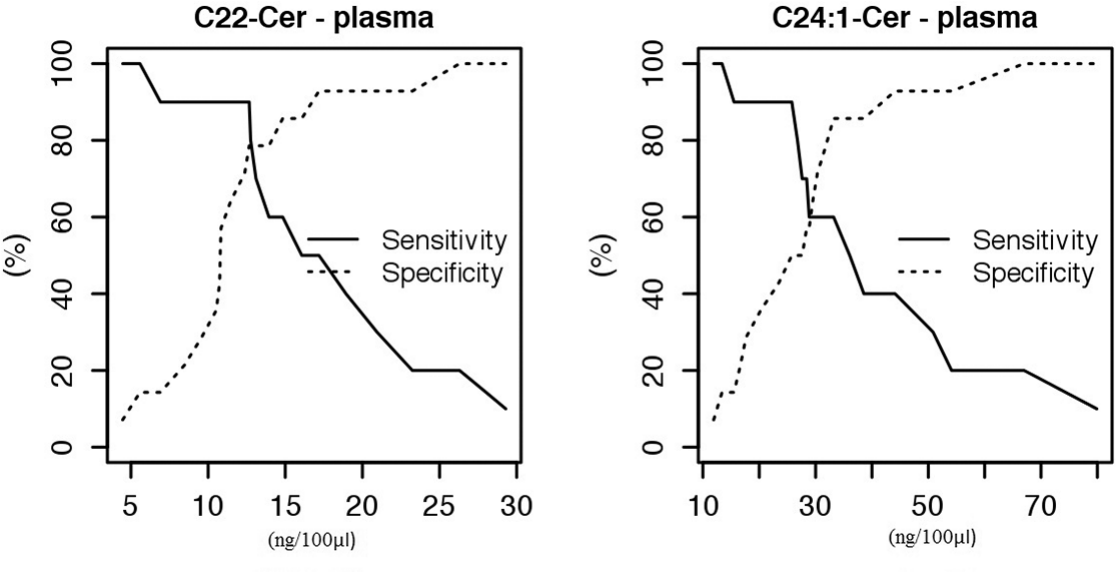
Sensitivity and specificity of biomarkers in plasma: C22-Cer and C24:1-Cer. We demonstrated a significantly higher risk of Down Syndrome when the plasma concentration of C22-Cer > 12.66 ng/100ul (sens. 0.9, sp. 0.79, *P value =* 0.0007) and C24:1-Cer > 33,19 ng/100ul (sens. 0.6, sp. 0.86, *P value =* 0.0194).

We demonstrated a significantly higher risk of Down Syndrome when the plasma concentration of C22-Cer > 12.66 ng/100ul (sens. 0.9, sp. 0.79, *P value =* 0.0007) and C24:1-Cer > 33,19 ng/100ul (sens. 0.6, sp. 0.86, *P value =* 0.0194) (Fig 2).

Diagnostic values of these sphingolipids in plasma and amniotic fluid are presented respectively in Table 5 and Table 6.

**Table 5:** Diagnostic values of sphingolipids in plasma.

	Threshold value (ng/100μl)	Sensitivity	95% CI for sensitivity	Specificity	95% CI for specificity	AUC	95% CI for AUC	Std. error	*P* value
C22-Cer	>12.66	0.9	0.5958–0.9821	0.7857	0.5241–0.9242	0.81	0.65–1	0.09	0.0007
C24:1-Cer	>33.19	0.6	0.3126–0.8318	0.8571	0.6005–0.9599	0.72	0.54–1	0.1	0.0194

**Table 6:** Diagnostic values of sphingolipids in amniotic fluid.

	Threshold value (ng/100μl)	Sensitivity	95% CI for sensitivity	Specificity	95% CI for specificity	AUC	95% CI for AUC	Std. error	*P* value
C16-Cer	<10.32	0.8888	0.565–0.9801	0.8571	0.6005–0.9599	0.8571	0.7183–1	0.08	<0.0001
C18:1-Cer	<0.015	1	0.7–1	0.7142	0.4535–0.8827	0.8968	0.7916–1	0.06	<0.0001
C18-Cer	<0.479	0.8888	0.565–0.9801	0.9285	0.6853–0.9872	0.9682	0.9185–1	0.03	<0.0001
C20-Cer	<4.52	1	0.7008–1	0.8571	0.6005–0.9599	0.9603	0.9003–1	0.03	<0.0001
C22-Cer	<6.95	0.6666	0.3542–0.8794	1	0.7846–1	0.873	0.7384–1	0.08	<0.0001
C24:1-Cer	<14.82	0.7777	0.4525–0.9367	1	0.7846–1	0.9047	0.7896–1	0.06	<0.0001
C24-Cer	<13.8	0.7777	0.4525–0.9367	0.8571	0.6005–0.9599	0.8015	0.6185–1	0.11	0.0033

We did not find any statistically significant differences when we compared plasma concentration of CRP between study and control group using Wilcoxon rank-sum test.

## Discussion

It is difficult to compare results of our investigation to any other research, because there are not many articles about profiling sphingolipids in maternal blood and amniotic fluid in patients with chromosomal abnormalities. Nevertheless, it is possible to associate some information existing in the scientific literature with our study results. There are potential explanations for the role of differentially expressed sphingolipids in the pathophysiology of Down syndrome.

The role of phospholipids and sphingolipids in various diseases which pathomechanisms are related to impaired myelination/demyelination of neurons in the brain, for example: Farber disease, Gaucher disease, Niemann-Pick disease, Alzheimer's disease and most importantly—Down syndrome have already been presented [25–28]. Murphy et. al. showed reduced content of sphingolipids in the brain tissue of people with Down syndrome [27]. This is associated with delayed myelination of neurons in the developing brain of children with trisomy of chromosome 21 resulting in mental retardation of these people [29]. In addition, other studies of neonatal brain showed (besides delayed myelination) fewer neurones, lower neuronal density and distribution as well as abnormal synaptic density and length, probably caused by abnormal neuronal migration in fetus and retarded synaptogenesis [30]. Engidawork et. al. noted that despite the absence of morphological changes in the structure of the brain of the fetus, there are several (described by them) biochemical and molecular changes in protein profile, that play a significant role in the development of morphological changes in the brain of newborns and children [29]. Similar changes as in the profile of proteins and genes encoding them, can occur in the lipid profile, specifically sphingolipid profile, in the brain of fetuses with Down syndrome, which may indicate the results obtained in our study.

Summing up the results of our study, we found reduced levels of seven ceramides (C16-Cer, C18-Cer, C18:1-Cer, C20-Cer, C22-Cer, C24:1-Cer, C24-Cer) in amniotic fluid and increased content of two ceramides (C22-Cer and C24:1-Cer) in plasma of pregnant women with fetal Down syndrome. These results relate quite well with recently published metabolomics study [12]. The researchers postulated that the increased levels of 3-hydroxybutyrate is the result of not sufficient usage of this metabolite in the process of delayed myelination of neurons in the brain of the fetus with trisomy of chromosome 21 [12, 13]. This hypothesis seems to be confirmed and complement by our results. Elevated concentration of unused (in myelination) metabolites results in the higher sphingolipids synthesis in the mother's body, which is proved by increased levels of two ceramides in our study. In addition, reduced levels of sphingolipids and metabolites in the fetus's body can indirectly provide a drastically reduced level of sphingolipids in the amniotic fluid of women with fetal Down syndrome in relation to the amniotic fluid of healthy pregnant women. The composition of the amniotic fluid may be somewhat reflection of the processes taking place in the body of the fetus and in order to verify this correlation of sphingolipids and metabolites is currently planned in our lab as part of the larger study that is going to be performed on amniotic fluid and plasma of patients with fetal Down syndrome.

The purpose of our study was to generate possibly new compounds that could contribute to elucidation of the pathogenesis of fetal Down syndrome and be potentially used as additional markers that could help to reduce false positive rates based noninvasive assessment. We realize that our results should be correlated with the other biochemical (double test), genetic (cell free fetal DNA) and ultrasound (nuchal translucency- NT) readouts that are obviously available, however, above mentioned test are routinely performed in 11th-14th weeks of gestation and blood samples for our sphingolipid analysis were taken after amniocentesis (15th – 18th weeks of gestation). Therefore correlation between biochemical, genetic and ultrasound together with sphingolipid analysis might not be reliably extrapolated which can be treated as a limitation of our study.

In this publication we showed that selected sphingolipids could be potential biomarkers of Down syndrome pregnancies and might play a role in the pathology of trisomy of chromosome 21. In the international literature there still exist no relevant research focused on the role of sphingolipids in the pathogenesis of Down syndrome. However, due to the complexity of the pathomechanism responsible for Down syndrome, further functional experiments should be performed.
